# Injectable Trimethyl Chitosan Hydrogel with Capsaicin Mitigates Functional Pain and Angio-Osteogenic Coupling in an Animal Model of Osteoarthritis

**DOI:** 10.34133/bmr.0424

**Published:** 2026-10-29

**Authors:** Kyung Wook Kim, Andrew Reyes Padalhin, Hyun Seok Ryu, Celine Abueva, Jun Sang Bae, So Young Park, Hong Bae Jeon, Ji-Hun Mo, Phil-Sang Chung, Hyun Sik Gong, Seung Hoon Woo

**Affiliations:** ^1^ Department of Orthopaedic Surgery, Dankook University Hospital, Dankook University College of Medicine, Cheonan, Republic of Korea.; ^2^ Department of Orthopedic Surgery, Seoul National University Bundang Hospital, Seoul National University College of Medicine, Seongnam, Republic of Korea.; ^3^ Beckman Laser Institute Korea, Dankook University College of Medicine, Cheonan, Republic of Korea.; ^4^ Dankook Institute of Medicine & Optics, Dankook University, Cheonan, Republic of Korea.; ^5^ Medical Laser Research Center, Dankook University, Cheonan, Republic of Korea.; ^6^ Department of Plastic and Reconstructive Surgery, Dankook University Hospital, Dankook University College of Medicine, Cheonan, Republic of Korea.; ^7^ Department of Otorhinolaryngology-Head and Neck Surgery, Dankook University Hospital, Dankook University College of Medicine, Cheonan, Republic of Korea.

## Abstract

Osteoarthritis (OA) remains a leading cause of disability worldwide, with current therapies limited to symptomatic relief. In this study, we developed and evaluated a thermoresponsive hydrogel system based on *N*,*N*,*N*-trimethyl chitosan (TMC) for the sustained intra-articular delivery of capsaicin, an established analgesic. This study focused on assessing the system’s biocompatibility, anti-inflammatory effects, and its impact on functional recovery in an animal model. A thermoresponsive hydrogel (1% TMC, 10% β-glycerophosphate) was formulated for stable gelation at 37 °C. Biocompatibility was assessed via cell count assays on rat articular chondrocytes and macrophages. Therapeutic impact was evaluated through rotarod performance and histological assessment of cartilage integrity in monoiodoacetate-induced OA in rats. Macrophage polarization was analyzed via immunofluorescence. In vitro, the TMC hydrogel successfully enhanced the biocompatibility of capsaicin while indicating a shift away from proinflammatory (M1) macrophage polarization, reflected in a 25% reduction of CD86^+^ cells while increasing CD206^+^ cells by 30%. Furthermore, this group exhibited consistent improvement in rotarod latency for up to 4 weeks post-treatment. Histopathological grading scores corroborated these functional improvements, with OA animal models treated with the TMC hydrogel loaded with capsaicin reaching only an average score of 2 compared to untreated control group’s 6. Immunofluorescence analysis revealed marked reduction in angiogenic and interleukin-6 inflammatory markers compared to control groups. The TMC–capsaicin hydrogel system shows promise as a biocompatible localized injectable treatment for OA. This system was associated with improved functional performance and joint protection, along with reduced inflammatory marker expression and preservation of cartilage tissue integrity.

## Introduction

Osteoarthritis (OA) is one of the most prevalent and chronic joint diseases, characterized by progressive structural deterioration involving the articular cartilage, subchondral bone, ligaments, joint capsule, and synovium, ultimately leading to the breakdown of cartilage and other periarticular tissues [1]. It is clinically characterized by presentation of chronic joint pain and stiffness, leading to mobility problems affecting one’s everyday activity. In 2020, OA affected approximately 7.6% of the global population, and its prevalence has continued to rise in association with aging societies [2]. Despite the extensive and long-standing global burden of OA, no definitive curative treatment has yet been established. Cartilage is particularly difficult to regenerate because it is hypocellular and avascular, and the limited repair that occurs usually results in inferior fibrocartilage [3]. Current therapeutic strategies for OA include lifestyle modification and pharmacological treatments aimed at pain relief and functional improvement, with surgical intervention as the last resort when these options fail [4].

Chitosan and its derivatives have emerged as promising candidates within the evolving research landscape for the development of advanced biomaterials for sophisticated drug delivery [5]. This natural polysaccharide, which can be extracted from crustaceans or mushrooms, is a linear polymer composed of *N*-acetylglucosamine and d-glucosamine units derived from deacetylation of chitin [6]. Despite its inherent advantages, chitosan is limited by its low solubility under normal physiological pH values of 6.5 and above. This pH-dependent solubility constrains its utility in certain applications that require normal physiological conditions [7]. This limitation can now be addressed by using a chemically modified chitosan—*N*,*N*,*N*-trimethyl chitosan (TMC). TMC is a highly cationic derivative synthesized through quaternization of the amino groups on the chitosan backbone [8,9], making it highly soluble in a wider pH range without losing the benefits [10]. Additionally, its permanent positive charge enhances mucoadhesion and permeability, thereby improving its interaction with cells and tissues [11].

Capsaicin, the active component of chili peppers, has attracted considerable interest for its analgesic and anti-inflammatory properties, which are particularly beneficial in the management of OA-related pain [12,13]. Capsaicin selectively binds to the vanilloid receptor subtype TRPV1 expressed on type C primary afferent fibers, leading to an increase in substance P release within the synaptic cleft [14]. These selective neuronal effects are particularly relevant in OA, where nociceptive fibers are densely distributed within periarticular and subchondral structures [15]. The efficacy of capsaicin in managing OA-related pain has been demonstrated in several studies [12], and it is widely approved as a topical therapy for the treatment of peripheral neuropathic pain. However, capsaicin has not yet been approved as an injectable therapy for the treatment of pain originating from deeper anatomical structures. Capsaicin’s use is limited by its poor water solubility, unwanted burning sensation, and high first-pass metabolism leading to repeated dosing [12,16]. This presents a challenge for its formulation requiring the management of initial acute burning sensation while ensuring prolonged exposure to reach proper localized analgesia.

This study aims to investigate the formulation, in vitro biocompatibility, and in vivo evaluation of a novel TMC hydrogel system specifically designed for the sustained and targeted delivery of capsaicin in the treatment of OA. We hypothesize that sustained release of capsaicin via hydrogel delivery may influence macrophage polarization and angiogenesis, thereby contributing to attenuated OA progression. The study will focus on its biocompatibility and anti-inflammatory effects, with particular attention to its impact on articular cartilage integrity and subchondral bone remodeling.

## Materials and Methods

### Optimization of capsaicin loading in TMC hydrogel

TMC chloride was synthesized and characterized based on a procedure adapted from Abueva et al. [8] Low molecular weight chitosan (50 to 190 kDa, derived from crustaceans, Sigma-Aldrich, USA) was utilized as the starting material to produce TMC. The synthesis involved *N*-methyl-2-pyrrolidone (NMP; Sigma-Aldrich, USA), sodium iodide (Merck Millipore, USA), and iodomethane (Sigma-Aldrich, USA). Purification was achieved through dialysis, followed by lyophilization to obtain the final product. Following previous studies on TMC thermoresponsive hydrogel formulation, stock solution was created by dissolving 4% TMC (w/v) in Dulbecco’s phosphate-buffered saline (DPBS) and 20% (w/v) β-glycerophosphate (β-GP) solutions. Referencing the results of Ryu et al. [17], the TMC hydrogel composition tested for this study was 1% TMC with 10% β-GP. This composition was selected because it can form a stable gel at 37 °C and has relatively better stem cell compatibility compared to formulations. Capsaicin (Sigma) was then dissolved in dimethyl sulfoxide (DMSO) at varying concentrations. The effect of DMSO on the gelling capability of the TMC hydrogel was then tested at different concentrations (% v/v) together with capsaicin at a fixed concentration of 250 μM. After mixing the TMC hydrogel with increasing concentrations of DMSO–capsaicin, 1 ml of each solution was carefully dispensed at an angle toward one side of separate wells in a 12-well plate and incubated at 37 °C for 5 min. After incubation, pictures of the samples were taken by tilting the plate toward the opposite side of the samples to evaluate whether a stable gel had formed and did not flow.

The temperature-dependent rheological properties of the TMC hydrogel (with and without capsaicin) were evaluated using a rotational rheometer (ARES-G2, TA Instruments, USA). A 40-mm parallel-plate geometry was used for the analysis. Each sample was subjected to a 60-s pre-shear step prior to data acquisition. The viscosity measurements were performed over a temperature range of 0 to 40 °C, with a constant heating rate of 5 °C/min and a fixed shear rate of 100 s^−1^. Measurements were also done to determine the effect of capsaicin loading in the TMC hydrogel at different temperatures. To evaluate the clinical utility and thermosensitive gelation behavior, the data analysis was focused on the physiological window of 25 to 37 °C, which encompasses the transition from ambient storage condition to body temperature.

The cumulative release of capsaicin from the hydrogel was measured through ultraviolet–visible (UV–vis) spectroscopy. In a 5-ml tube, 500 μl of the TMC hydrogel containing either 50, 250, or 500 μM capsaicin was pre-gelled, after which 3.0 ml of PBS with 10% ethanol was added as the release medium. A separate tube containing the TMC hydrogel with an equal amount of DMSO without capsaicin was also prepared as a blank reference for calculation. The sealed tubes were then placed inside a 37 °C incubator. At designated time intervals (4, 8, 12, and 24 h, followed by daily reading for 1 week), a 600-μl aliquot of the release medium was sampled from each tube and plated in a UV-transparent 96-well plate (SPL, UVMax 33096). To maintain the proper volume of release medium, each tube was replenished with an equivalent amount of release medium. Absorbance was read at 281 nm using a Tecan Infinite M200 Pro microplate reader. Cumulative capsaicin was calculated by subtracting the absorbance reading from the blank and converting the amount based on previously established standard curve while accounting for the progressive dilution from sampling.

### In vitro cytocompatibility of capsaicin-loaded TMC hydrogel cell viability testing

A modified method for harvesting rat articular chondrocytes (RACs) was carried out as previously described [18]. RACs were extracted from the articular cartilage of 3-week-old male Sprague-Dawley rats. The animals were euthanized via an overdose of anesthesia, and knee cartilage was carefully removed, sliced into thin pieces, and washed with sterilized PBS. Tissue slices were soaked in 5% penicillin–streptomycin–neomycin (PS) solution (Sigma, St. Louis, MO, USA) for 15 min and then washed again with PBS to eliminate any residual antibiotics. The cartilage slices were digested in 0.02% collagenase type II (Sigma) dissolved in Dulbecco’s modified Eagle’s medium (DMEM; HyClone, Logan, UT, USA) for 3 h in a 37 °C water bath. Following digestion, the suspension was centrifuged, and the resulting pellet was resuspended in DMEM and filtered through a 70-μm nylon mesh. The isolated chondrocytes were then cultured in DMEM supplemented with 10% fetal bovine serum (FBS) and 1% PS and maintained in a 5% CO₂ incubator at 37 °C. Macrophage cells (RAW264.7) were purchased from the American Type Culture Collection and maintained with DMEM containing 10% FBS and 1% penicillin–streptomycin.

Cell viability assays were performed in a 96-well plate seeded with 1,000 cells/well and incubated overnight at 37 °C with 5% CO₂ to allow adherence. The medium was then replaced with fresh medium containing increasing concentrations of capsaicin or 10% volume of the TMC hydrogel with increasing capsaicin content. Untreated cells and blank wells containing no cells were used as controls. After 24 h, the medium was replaced with 90 μl of fresh medium containing 10 μl of the Cell Counting Kit-8 (CCK-8; Dojindo Laboratories, Kumamoto, Japan) reagent and incubated for another 2 h. The absorbance at 450 nm was measured using a microplate reader. The absorbance data were analyzed by subtracting blank reading and calculating cell viability relative to untreated controls.

### Macrophage polarization test

Immunofluorescence (IF) staining was conducted to examine the effect of capsaicin, TMC hydrogel, and TMC hydrogel containing capsaicin on macrophage polarization. Macrophage cells (RAW264.7) were seeded in individual wells of a 96-well plate at a density of 10,000 cells/well. After complete adherence, RAW264.7 cells were incubated in DMEM containing 1.0 μg/ml of *Escherichia coli* lipopolysaccharide (LPS; SMB00801-25MG, Sigma-Aldrich, Missouri, USA) with test samples for 24 h. After complete incubation, the medium was removed from each well and washed twice with PBS. The cells were then fixed with 4% paraformaldehyde for 10 min and incubated with rabbit anti-mouse CD86 (PA5-88284, Invitrogen, California, USA; dilution 1:200) and goat anti-mouse CD206 (PA5-46994, Invitrogen, California, USA; dilution 1:100) primary antibodies overnight at 4 °C followed by goat anti-rabbit Alexa Fluor 594 (A11058, Invitrogen, California, USA; dilution 1:200) and donkey anti-goat Alexa Fluor 488 (A27012, Invitrogen, California, USA; dilution 1:200) and then stained with 10 μg/ml 4′,6-diamidino-2-phenylindole (DAPI) for 10 min. Micrographs were then taken using a confocal microscope (FV-3000; Olympus, Tokyo, Japan). Images were then analyzed using QuPath [19] opensource software (version 0.7.0) to determine cells positive for CD86, CD206, or both. The threshold range for cell detection based on nuclear DAPI staining was first established using 10 randomly selected images spanning all treatment groups. Threshold ranges for positive marker detection were then defined using representative images from groups with known or visible high marker expression (3 images from the +LPS group for CD86; 3 images from TMC hydrogel + Capsaicin for CD206). These threshold values for detection were then fixed prior to quantification and applied uniformly to all images across all groups in a single batch run. Because threshold calibration was determined using known group identity, investigators were not blinded during the analysis. Data were normalized based on the cell count and presented as percentage positive for either marker.

### Animal experiments

All procedures for this experiment were evaluated, approved, and conducted according to the rules and regulations set by the Institutional Animal Care and Use Committee of Dankook University (DKU-23-083). A total of 48 six-week-old male Sprague–Dawley rats with an average weight of 250 g were obtained from Orient Bio (Songnam, South Korea). The animals were housed in pairs and provided with ad libitum food and water. The rats were acclimatized in a climate-controlled animal room with a 12-h light–dark cycle for 1 week prior to chemical induction of the OA and further experimentation.

OA was induced in the animals by intra-articular injection of monoiodoacetate (MIA) based on previous studies on chemically induced OA animal model [20]. MIA (Sigma-Aldrich, St. Louis, MO) was dissolved in saline at a concentration of 0.01 mg/μl. The animals were anesthetized using isoflurane inhalation in an induction chamber. Upon full anesthetic induction, the animal was positioned supine on operating table and the fur was shaved from both knees to expose the skin surface, which was then sterilized with 70% ethanol. The injection site was located by bending the knee joint at a 90° angle tracking the patellar ligament of the tibia and identifying the soft spot along the medial region of joint. Using a 300-μl tuberculin syringe, a 50-μl solution containing 0.5 mg of MIA was injected within the intra-articular space of both left and right knee joints. The animal was then returned to its home cage to recover fully. Animal weight was monitored throughout the duration of experiment to ensure animal welfare and health (Fig. S1).

### Forced ambulatory performance test

Rotarod test was used to determine the effect of the chemically induced OA on the functional condition of the animals’ knee. The rotating rod creates forced motor activity to evaluate knee function using latency (seconds), which ends when the animal falls off. Each animal was trained for 5 min at a constant speed of 4 rpm on the rotarod 3 times a day for 3 d. The animals are then allowed to have a 2-day break before the first trial. To establish the baseline reading, the animals are first re-acquainted with the rotarod activity for 5 min at a continuous speed. The first trial is then initiated by switching to an accelerated speed of 30. Each animal is tested at least 3 times, with each test having an initial rest time of 20 min by rotating each group per trial. Subsequent rotarod trials are then performed once a week until tissue sampling. Reduction of performance (decreased latency time on the rotarod) is used as a measure of decline in joint function due to MIA-induced OA.

### Treatment application in OA animal model

The therapeutic effect of the different treatments was tested upon confirmation of the initial decline of all MIA-treated animals in the rotarod test (2 weeks post-OA induction). Briefly, the animals are anesthetized with isoflurane inhalation and a bilateral injection of the treatment materials was used based on designated groupings. The rats were randomly selected into 6 groups (4 rats per group) and 2 observation points (2 weeks post-treatment and 4 weeks post-treatment). All groups aside from the negative control were subjected to bilateral injection of MIA during the OA induction phase of the experiment. The 6 groups are as follows: Negative control—animals injected with saline during OA induction and injected with saline as treatment; Positive control MIA-OA group—injected with saline as treatment; TMC group—injected with the TMC hydrogel as treatment; Capsaicin group—animals injected with capsaicin as treatment; TMC-Cap group—animals injected with the TMC hydrogel with capsaicin as treatment; and Polynucleotide (PN) group—animals treated with a commercial intra-articular injectable PN with hyaluronic acid (Conjuran, Pharma Research, South Korea), a common and rapidly growing treatment for knee OA in South Korea. It is known to provide localized biological tissue support and affect inflammatory pathways, making it a suitable commercial comparator for comparing inflammatory markers for managing disease progression. All treatments were injected at a volume of 50 μl per injection site. Post-injection, the animals were regularly monitored for signs of severe distress (weight loss). Routine rotarod testing was performed in all rats once a week for 2 and 4 weeks post-treatment injection, after which the designated animals were sacrificed and tissue samples were extracted and processed for further analysis.

### Macroscopic observation of joint integrity

The animals were sacrificed at 2 and 4 weeks post-treatment. Tissue samples were extracted by exposing the knee tissue and dislocating the distal femoral head. Surrounding connective tissue and muscle were dissected from the shaft of the femur before cutting off the distal femur head using a circular saw blade. Tissue samples were fixed in a 10% neutral buffered formalin. Before imaging, tissue samples are cleared of surrounding connective tissues and mounted on a plastic holder with a white background. A solution of 5% India ink was brushed over the tissue sample to enhance the contrast, and excess ink was removed by slightly dabbing with tissue paper. Images of the surface of the distal femur head were taken using a dissecting microscope with camera attachment.

### H&E and Safranin-O fast green staining

Tissue decalcification was performed using a 10% (wt/v) EDTA solution at room temperature for 2 weeks. The decalcified tissues were then processed for paraffin embedding using an automated Tissue-Tek VIP 5 Jr. processing machine (Sakura Finetek, USA). Paraffin-embedded tissue blocks were cut into 5.0-μm-thick sections using a Leica RM2135 microtome (Leica, Germany). Tissue sections were stained with hematoxylin and eosin (H&E) to highlight cellular and extracellular structures and Safranin-O fast green for visual emphasis of hyaline cartilage. Images of the tissue sections were taken using an Olympus BX53 light microscope equipped with cellSens imaging software (Build 18987). Images of the medial femoral condyle were analyzed for cartilage tissue thickness using FIJI software (ImageJ 1.54p, NIH, USA), and OA grade scoring for medial femoral condyle was adapted from the Osteoarthritis Research Society International (OARSI) histopathology initiative scoring protocol for mouse [21].

### IF imaging angiogenesis and osteogenesis coupling

IF staining was conducted to examine the presence of H-type blood vessel within the subchondral bone and expression of noggin and proinflammatory cytokine interleukin-6 (IL-6). For antigen retrieval in the deparaffinized and rehydrated tissue sections, a heat-induced antigen retrieval method was employed using citrate buffer (10 mM, pH 6.0). The tissue sections were then blocked with 3% bovine serum albumin to minimize nonspecific antibody binding. Individual slides were incubated with CD31 (AD3628, R&D Systems, Minnesota, USA; dilution 1:100) and endomucin (PA5-115178, Invitrogen, California, USA; dilution 1:200) primary antibodies for identification of H-type blood vessels and Noggin (BS-2975R, Bioss, Massachusetts, USA; dilution 1:200) and IL-6 (PA5-144595, Invitrogen, California, USA; dilution 1:200) primary antibodies to determine the effect of the treatments on cartilage integrity and tissue inflammatory response. Each primary antibody was paired with contrasting secondary antibody Alexa Fluor 594 and 488, respectively. IF-stained tissue sections were then counterstained for cellular nucleus and mounted with Vectashield with DAPI (H-1200, Vector Laboratories, Burlingame). Images were then acquired using a confocal microscope (FV3000; Olympus, Tokyo, Japan), and relative expression was quantified from the mean fluorescence intensity, averaged across 3 images per sample, using FIJI software (ImageJ version 1.54p).

### Statistical analyses

Animal number was determined using the “resource equation” method based on analysis of variance (ANOVA). The value of “*E*” is calculated by subtracting the number of groups from the total number of animals that should fall between 10 and 20 to be considered adequate. There were no criteria for inclusion/exclusion of animals during the experiment aside from maintaining the groupings. Statistical analyses for comparing the results of the cell viability and macrophage polarization tests made use of one-way ANOVA, while rotarod data, OARSI scores, and histological measurement data were analyzed using two-way ANOVA. These analyses were followed by the either Tukey, Dunnett, or Sidak post-hoc tests for comparison with the control or multiple comparisons among groups. Pearson’s correlation analysis was also conducted to determine the linear relationship between functional performance and expression of biomarkers. All statistical analyses and graphs were done using GraphPad Prism 9 (version 9.5.1, USA). Results are reported as mean values ± 95% confidence intervals (CIs), and a threshold value of *P* < 0.05 was applied to determine statistical significance in all analyses.

## Results

### Hydrogel optimization, cytotoxicity, and macrophage polarization

Cell viability shows that RACs tolerate at most 250 μM capsaicin (95% CI of difference [−14.07%, 12.07%], *P* = 0.9997), while macrophages can tolerate up to 125 μM capsaicin (95% CI of difference [−11.17%, 4.834%], *P* = 0.8318). Significant decline in cell viability was observed as exemplified in macrophage viability in which 500 μM (95% CI of difference [−78.17%, −62.17%], *P* < 0.0001) and 1,000 μM (95% CI of difference [−88.33%, −72.33%], *P* < 0.0001) indicate that these concentrations are not suitable for biological application (Fig. 1A). Based on this, a fixed concentration of 250 μM was used to optimize the amount of DMSO within a total volume of the hydrogel. The TMC hydrogel containing 1% TMC and 10% β-GP was used as the main formulation for testing with various concentrations of DMSO. Figure 1B shows the effect of increased DMSO content on the gelation property of the TMC hydrogel. Results indicate that DMSO composition higher than 10% severely prevents the formation of a stable gel incubated at 37 °C, while concentrations lower than 2.5% resulted to a similar gelation potential with that of the control TMC hydrogel (Fig. 1B). Viscosity measurements (Fig. 1C) confirm that addition of DMSO and capsaicin did not alter the effect of temperature (25 to 37 °C) on the gelling capacity of the resulting material. Compared to the TMC solution, the TMC hydrogel with or without capsaicin gradually increases in viscosity relative to increased temperature. Cumulative release profiles (Fig. 1D) indicate that the TMC hydrogel loaded with 500 μM capsaicin exceeded 50% release after 24 h and peaked at 72 h. While both 50 μM and 250 μM formulation showed similar release kinetics, the lower load concentration demonstrated 18% higher cumulative release at the end of the observation period.

**Fig. 1.**
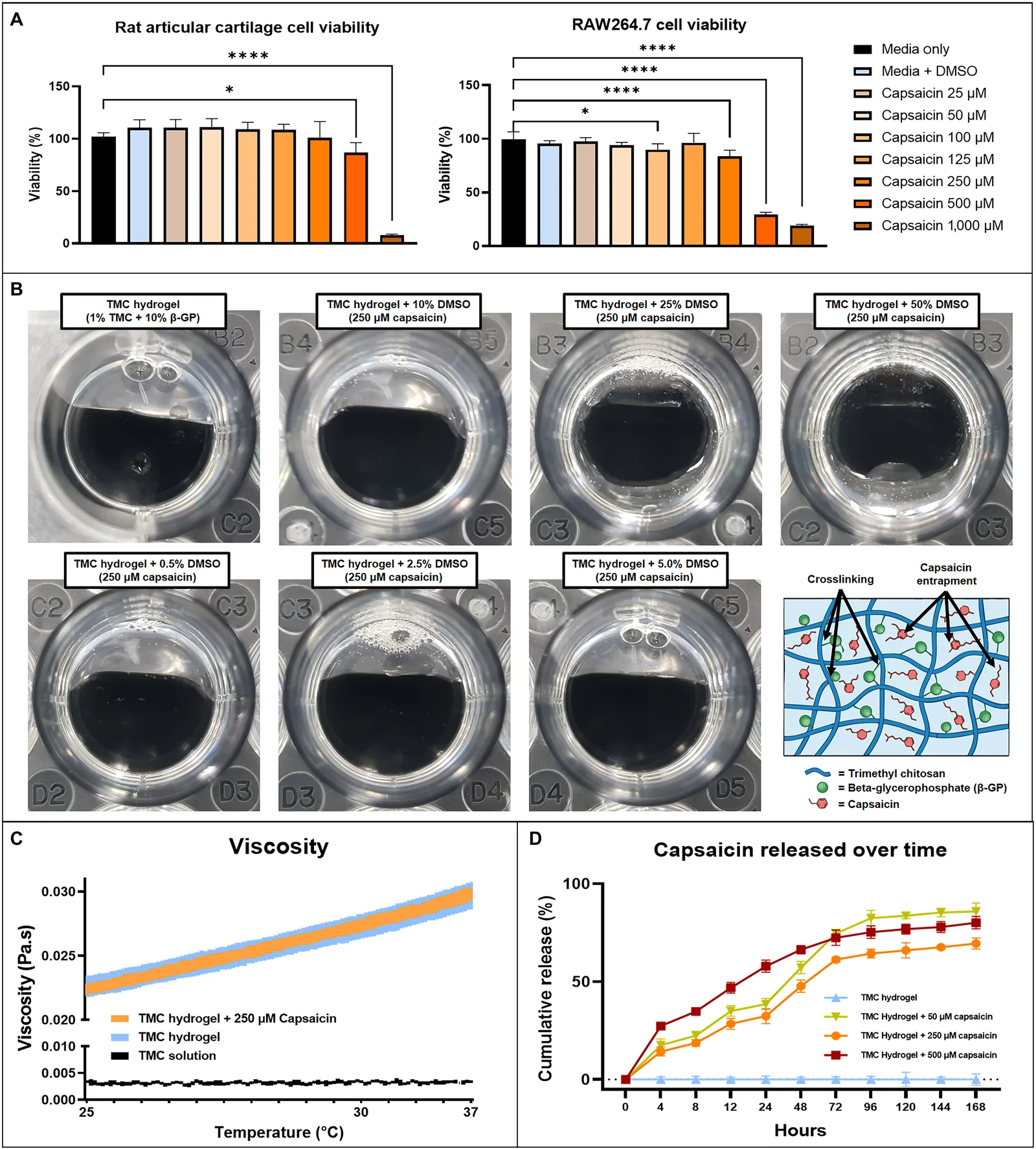
In vitro test determining tolerable concentration of capsaicin by rat articular cartilage and macrophage cells. (A) Results indicate that these cells can tolerate up to 250 and 125 μM capsaicin. (B) Different TMC hydrogel formulations with varying amounts of DMSO, indicating that the amount of DMSO affects the gelation capability of the TMC hydrogel. (C) Viscosity measurements showed no change in rheological properties in the TMC hydrogel upon loading with capsaicin. (D) Different formulation showed variable release kinetics, with high capsaicin load (500 μM) releasing 50% in just 24 h. One-way ANOVA with Dunnett’s multiple comparison test; *n* = 6. *P < 0.05, ****P < 0.0001.

Based on these observations, capsaicin can be loaded with less than 1% DMSO composition in the TMC hydrogel. The incorporation of capsaicin into the TMC hydrogel markedly improved its cytocompatibility profile compared to the free chemical. While free capsaicin at 500 μM induced slight and high toxicity to chondrocytes (86.6 ± 9.6% viability) and macrophages (29.1 ± 2.2% viability) (Fig. 1A), treatment with the TMC hydrogel containing 250 μM concentration maintained higher viability (>80%) for both RAC (95% CI of difference [−12.21%, 8.272%], *P* = 0.9932) and macrophage cells (95% CI of difference [−10.50%, 13.58%], *P* = 0.9984) (Fig. 2A), demonstrating the tempering effect of the hydrogel matrix on capsaicin toxicity. IF-stained proinflammatory macrophages (CD86-positive) were effectively suppressed by the combination of the TMC hydrogel and capsaicin. The visible increase in CD206-positive macrophages indicates that the TMC hydrogel and capsaicin mitigated proinflammatory polarization of macrophages activated with LPS (Fig. 2B). Results of the image analysis through QuPath positive cell counting further confirmed the effects of the individual treatments. While all treatment groups showed notable increase in CD206-positive cells, treatment with the TMC hydrogel alone did not significantly reduce expression of CD86 compared to LPS (95% CI of difference [−29.37%, 6.188%], *P* = 0.3389). In contrast, both capsaicin (95% CI of difference [−70.35%, −34.79%], *P* < 0.0001) and TMC hydrogel + 250 μM capsaicin (95% CI of difference [−48.50%, −12.94%], *P* = 0.0002) treatments resulted in significant reduction in CD86-expressing cells.

**Fig. 2.**
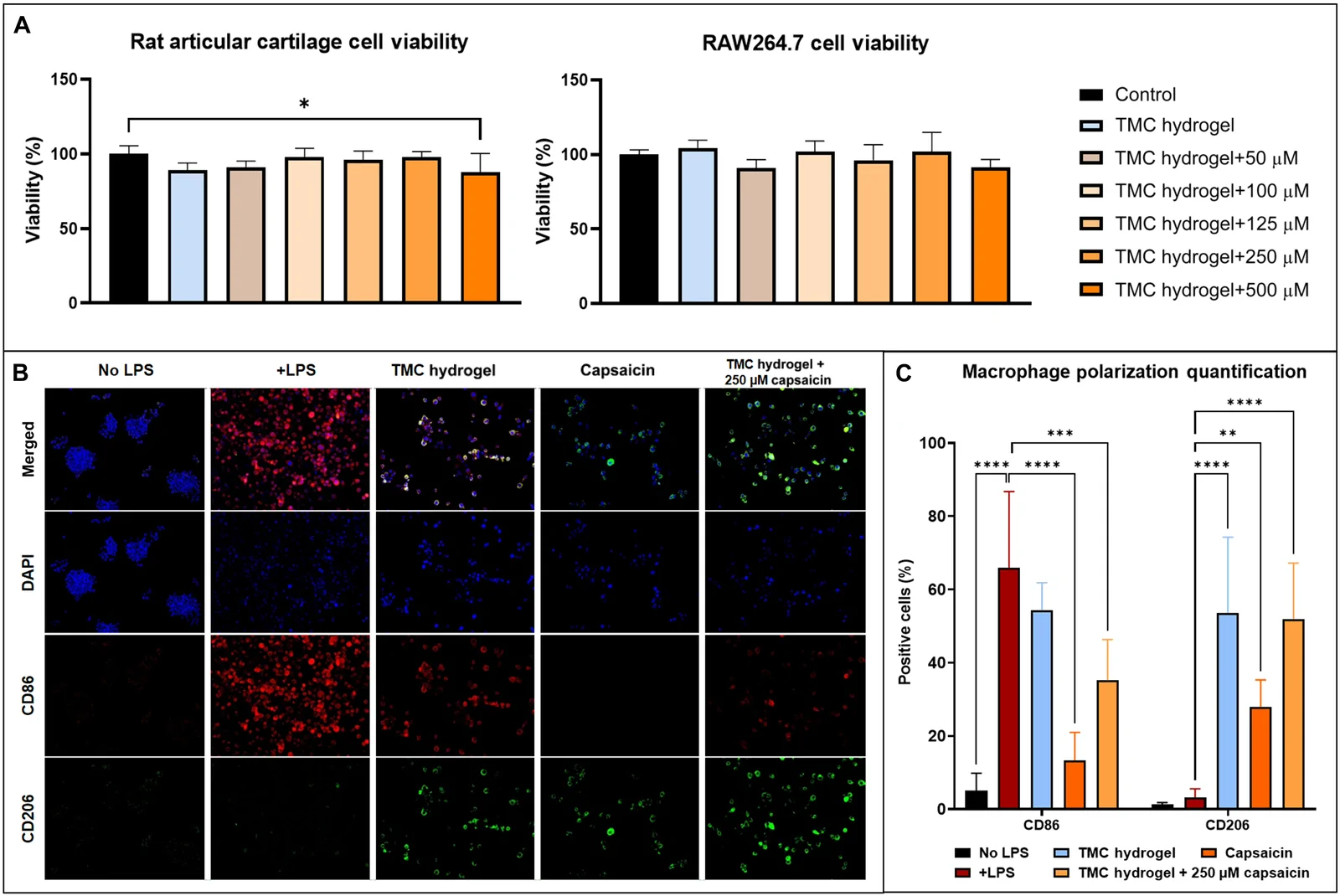
In vitro cytocompatibility and macrophage polarization effect of capsaicin-loaded TMC hydrogels. (A) Viability of both articular cartilage cells and macrophage cells in the TMC hydrogel containing varying concentrations of capsaicin shows similar tolerances. (B) In vitro micrographs of macrophage cells stained for CD206 (M2 marker) and CD86 (M1 marker) indicate that the combination of the TMC hydrogel and capsaicin mitigates proinflammatory polarization of macrophages activated with LPS. (C) QuPath quantification shows that the capsaicin-loaded TMC hydrogel simultaneously lowered CD86 and increased CD206 expression. One-way ANOVA with Dunnett’s multiple comparison test; *n* = 6. Two-way ANOVA; Sidak’s multiple comparison tests; *n* = 6. *P < 0.05, **P < 0.01, ***P < 0.001, ****P < 0.0001.

### Animal modeling of OA with functional testing

Figure 3A outlines the schedule of animal experiment conducted using chemically induced OA animal model with forced ambulatory testing. Results indicate that the bilateral intra-articular injection of MIA in rats resulted in a noticeable decline in rotarod latency after 2 weeks (Fig. 3B), which gradually worsens over succeeding observation periods. Histological images (Fig. 3C) of the distal femoral head showed substantial erosion of the cartilaginous layer of the joint over the course of 14 d. Thickness measurements (Fig. 3D) showed consistent decrease of cartilage thickness at 14 d (95% CI of difference [−170.2, −29.65], *P* = 0.0140), which coincides with the observed decline in rotarod performance within the same observation period of 2 weeks. With these results, the 2-week post-MIA injection was designated as the treatment point taking into consideration that most OA conditions are diagnosed upon presentation of symptomatic pain.

**Fig. 3.**
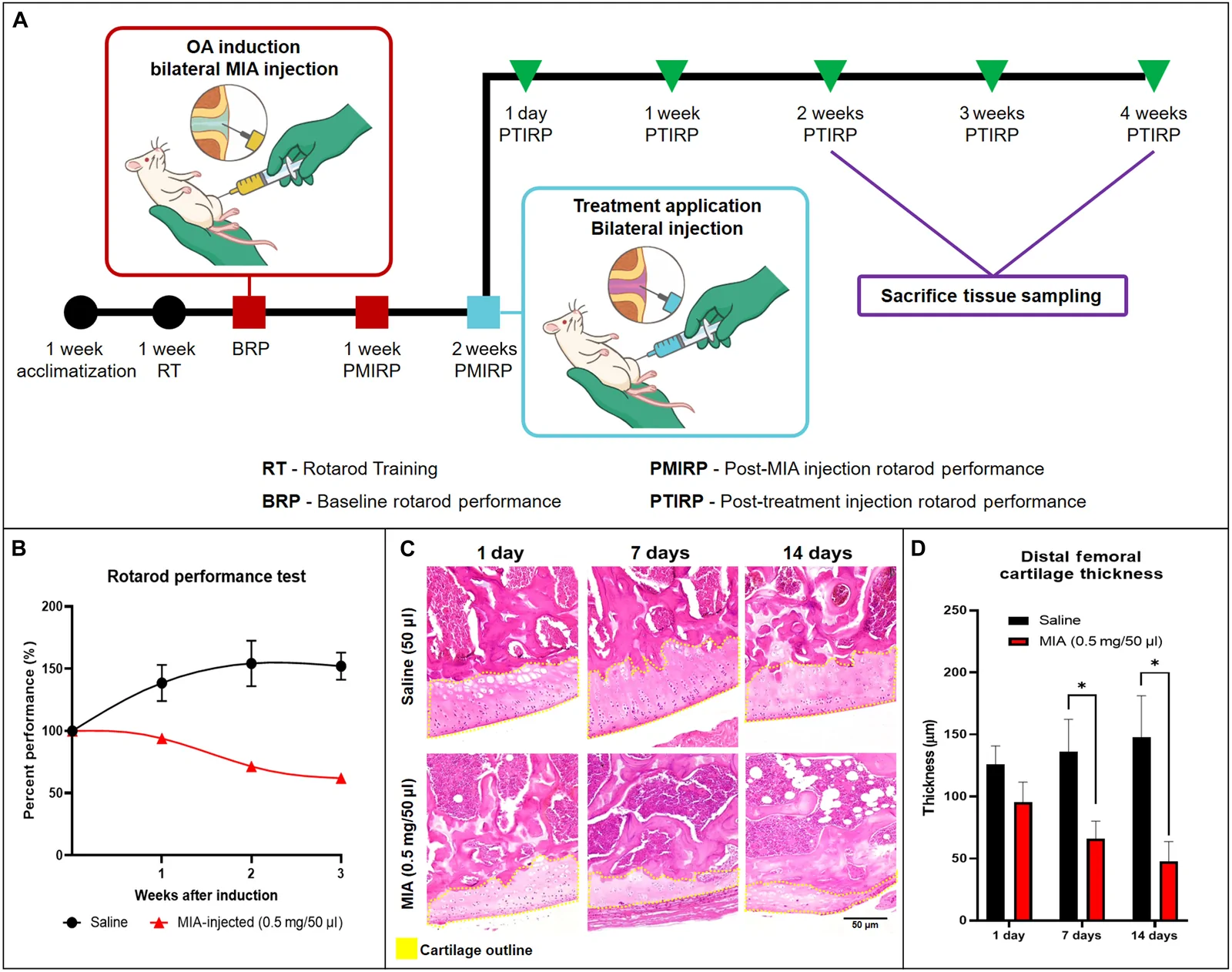
Experimental timeline and characterization of MIA-induced OA. (A) Schematic diagram showing the schedule and rotarod testing timeline. (B) Rotarod testing showed decreased performance, while histological images showed progressive tissue damage (C) and reduced cartilage thickness (D) following intra-articular injection of 0.5 mg/50 μl of MIA. Two-way ANOVA followed by Sidak’s multiple comparison test; *n* = 4. *P < 0.05.

### Post-treatment joint function and macroscopic integrity

After OA induction, treatment was injected into designated groups [MIA-Saline, MIA-Capsaicin (250 μM/50 μl), MIA-TMC hydrogel (50 μl), and MIA-TMC hydrogel (50 μl) + Capsaicin (250 μM); Fig. S2]. The same rotarod test was performed 1 day and 4 consecutive weeks after injection of the respective treatments (Fig. 4). Tissue sampling was conducted at second and fourth weeks of treatment. Functional assessment via the forced ambulatory rotarod test validated the subsequent performance between the negative control and the positive control (MIA group), showing subsequent improvement and decline of each group. All animals injected with MIA showed decline in performance 2 weeks after OA induction. The same trend was observed until 1 week post-injection of respective treatments per group. At 3 weeks post-injection of the treatment, all treatment groups showed marginal improvement to the MIA group. The rats that received the TMC hydrogel, capsaicin, and PN had a comparable latency time compared to the group treated with the TMC hydrogel with capsaicin. The same trend was also observed at 4 weeks post-treatment with the TMC hydrogel, with the capsaicin group performing consistently longer latency times (95% CI of difference [49.40, 109.9], *P* < 0.0001), while the performance of other groups declined (Fig. 4A). Macroscopic imaging of the extracted distal femur head revealed that capsaicin primarily promoted extensive fibrous tissue formation. Additionally, the TMC hydrogel with capsaicin appears to be better compared to the other treatments at 4 weeks post-treatment. However, from the perspective of cartilage preservation, PN-treated samples showed less degeneration compared to other groups (Fig. 4B).

**Fig. 4.**
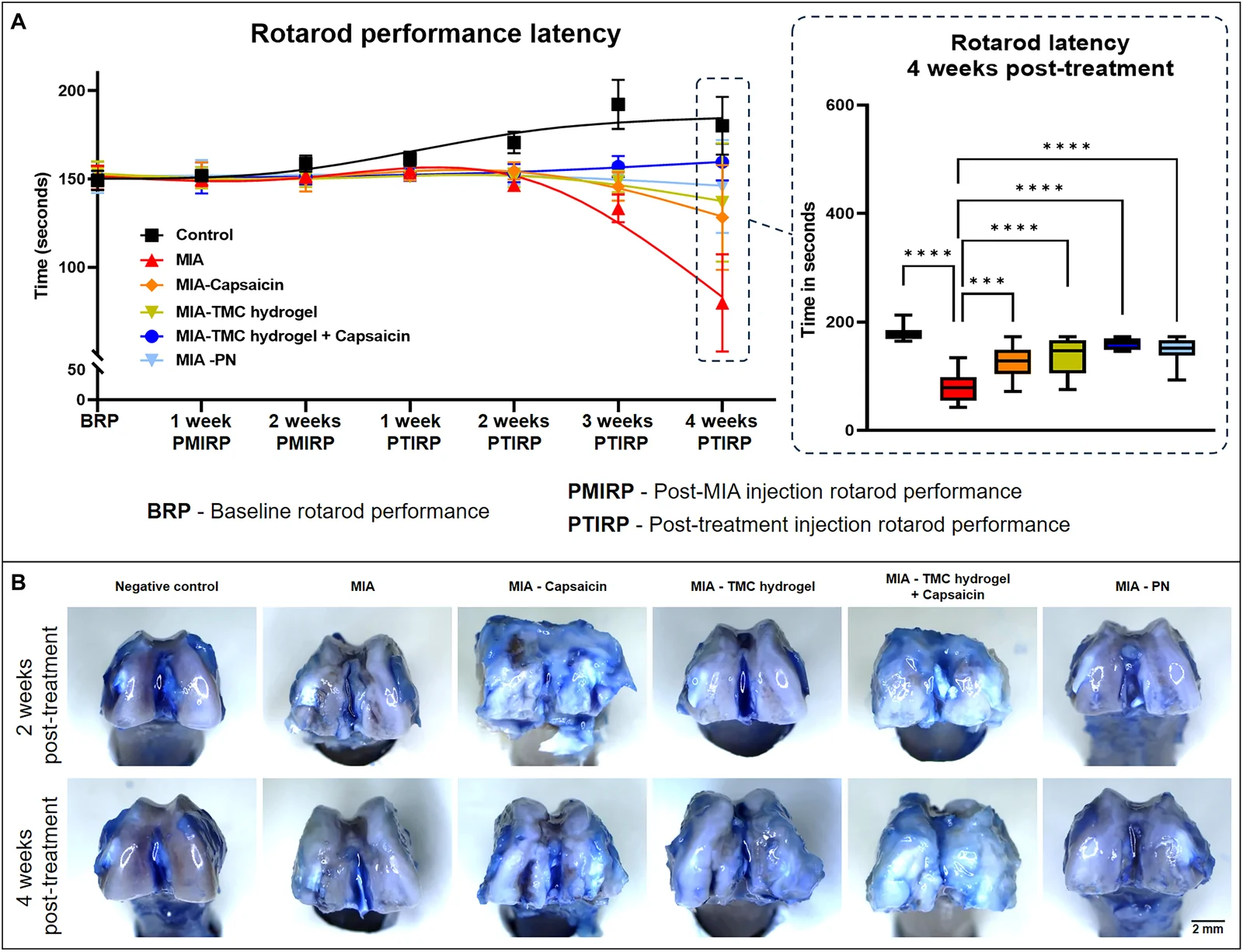
Therapeutic evaluation of capsaicin-loaded TMC hydrogels via forced ambulatory testing and macroscopic joint imaging. (A) Rotarod performance of the different treatment groups over the course of the OA induction up to 4 weeks post-treatment. (B) Representative macro-images of rat distal femur head stained with India ink to visualize articular cartilage damage. One-way ANOVA followed by Tukey’s multiple comparison test; *n* = 4. ***P < 0.001, ****P < 0.0001.

### Histological analyses

Histological evaluation using H&E and Safranin-O fast green staining confirmed the effects of the treatments on cartilage tissue integrity (Fig. 5A and B). Untreated MIA-induced OA showed chondrocyte clustering along the cartilage layer of the sampled tissue accompanied by the formation of pockets of vascularization (Fig. 5A, MIA). Treatment with capsaicin reduced chondrocyte clustering accompanied by hypertrophic chondrocytes but still showed formation of vessels. The TMC hydrogel and PN treatment showed substantial cellularity along within the thickened cartilage area. Intra-articular injection of the TMC hydrogel containing capsaicin mitigated the formation of blood vessels and reduced the formation of chondrocyte clusters without the presence of hypertrophic chondrocytes along the subchondral bone. Further degeneration of the cartilage layer was observed at 4 weeks post-treatment, with the MIA and Capsaicin group showing substantial damage with extensive fibrous tissue formation along the supposed cartilage region. The TMC hydrogel treatment resulted in the thick formation of cartilage area with clustered chondrocytes and hypertrophic chondrocytes along the subchondral bone region. This similar effect was also observed in the group treated with the TMC hydrogel containing capsaicin but with less chondrocyte clustering. Averaged OARSI histopathological grade scores (Fig. 5C) from the medial femoral condyle sections of the tissue samples showed marginal differences among the treatment groups at 2 weeks post-treatment. This was not the case at 4 weeks post-treatment, in which differences in tissue morphology were more pronounced particularly with the MIA group (95% CI of difference [−5.609, −4.141], *P* < 0.0001) (compared to control), scoring significantly higher compared to the TMC hydrogel (95% CI of difference [−4.224, −1.776], *P* = 0.0004), TMC hydrogel with capsaicin (95% CI of difference [−5.224, −2.776], *P* < 0.0001), and PN-treated groups (95% CI of difference [−5.903, −2.347], *P* = 0.0005). Among the groups, the MIA group exhibited extensive cartilage erosion and proteoglycan loss, whereas the TMC hydrogel alone provided notably improved cartilage preservation compared to the other groups. Moreover, capsaicin showed the poorest cartilage preservation results. Cartilage thickness measurements also further confirm the differences among the treatment groups. After 4 weeks, both the TMC hydrogel alone (95% CI of difference [277.5, 334.9] μm, *P* < 0.0001) and the TMC hydrogel combined with capsaicin (95% CI of difference [306.2, 363.6] μm, *P* < 0.0001) groups significantly showed superior cartilage preservation compared to the MIA group. Additionally, the TMC hydrogel + capsaicin group tended to exhibit greater cartilage thickness than the TMC hydrogel alone group (Fig. 5D).

**Fig. 5.**
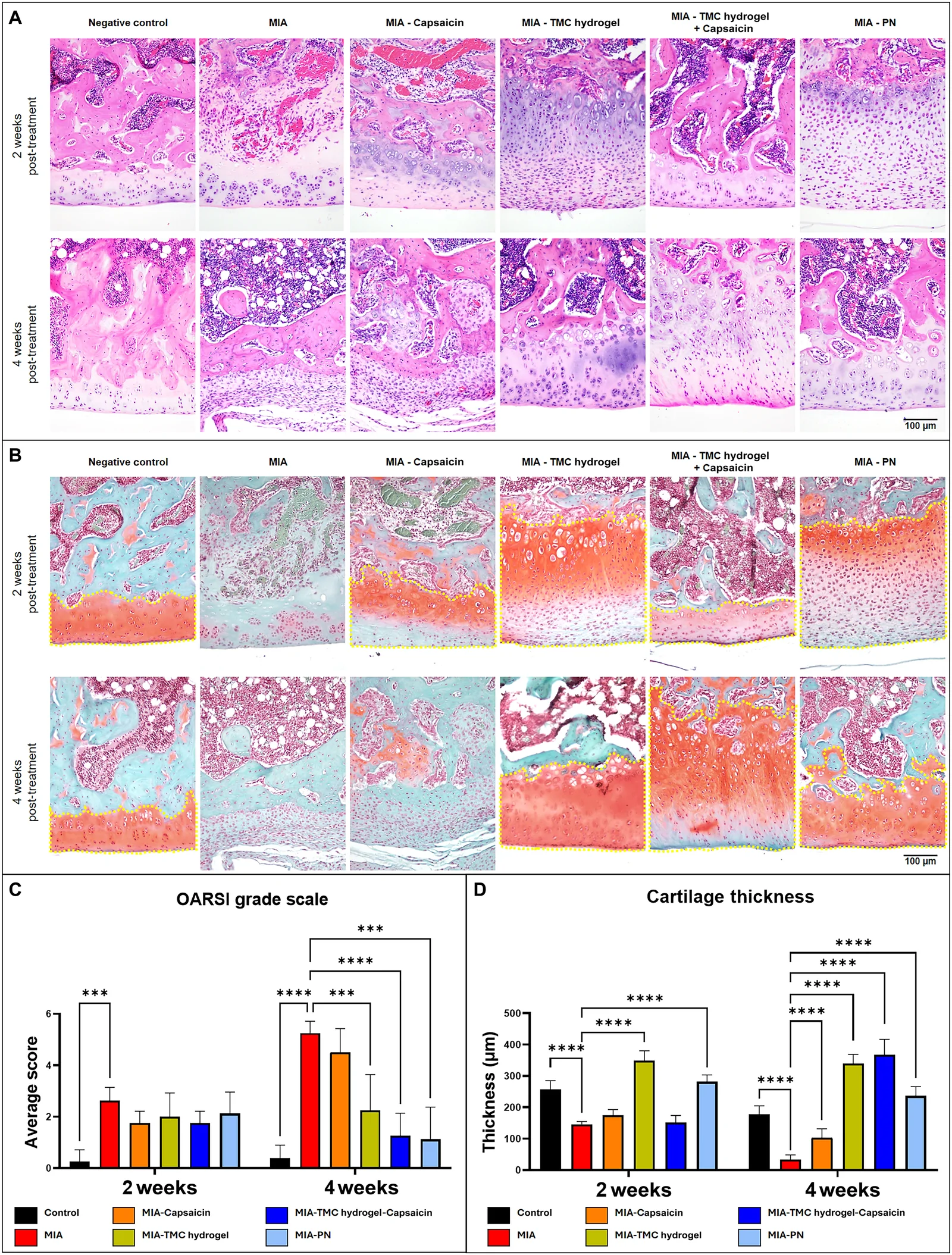
Histological assessment of joint pathology and cartilage thickness across treatment groups. Representative tissue sections stained with H&E (A) and Safranin-O fast green (B) showing the progression of chemically induced OA. (C) Average OARSI histopathological grade scale scores show markedly reduced tissue damage in treated groups 4 weeks post-treatment. (D) Measurements of cartilage thickness indicate variable results among different treatment groups. Two-way ANOVA followed by Dunnett’s multiple comparison test; *n* = 4 (3 images per sample). ***P < 0.001, ****P < 0.0001.

### Observation of angiogenesis and osteogenesis

IF staining was conducted to examine the presence of H-type blood vessel within the subchondral bone and expression proinflammatory cytokine to evaluate inflammation and cartilage protection. Increased expression of CD31 and endomucin, markers of H-type vascularization, in MIA-injected joint tissue shows irregular development of blood vessels within the subchondral bone tissue (Fig. 6A). Significant reduction in CD31 expression was observed across most treatment groups compared to MIA, apart from the TMC hydrogel group (95% CI of difference [−46.00, 0.633], *P* = 0.0507) (Fig. 6B). However, only TMC hydrogel with capsaicin (95% CI of difference [−83.40, −9.674], *P* = 0.0110) was effective in reducing expression of endomucin, indicating lower angiogenesis in the subchondral bone (Fig. 6B). Further analysis of these biomarkers with the rotarod latency indicated strong to moderate negative correlation, demonstrating that the presence of H-type blood vessels can negatively impact functional performance in osteoarthritic knee joint (Fig. 6C).

**Fig. 6.**
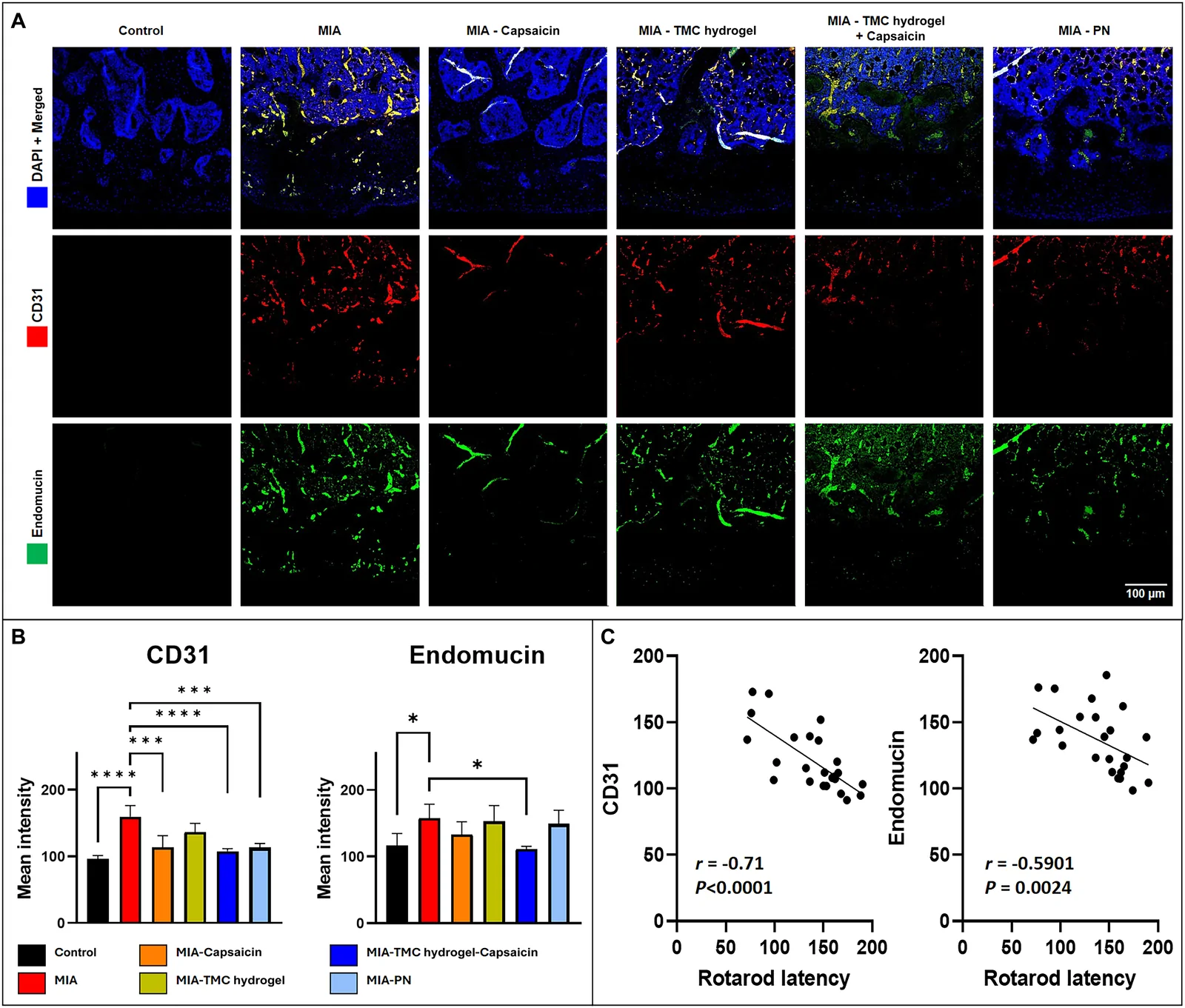
(A) Micrographs of tissue sections stained for CD31 and endomucin to observe the effect of the different treatments on cartilage tissue vascularity. (B) Mean intensity measurements of CD31 and endomucin expression from confocal images. (C) Pearson’s correlation analyses between the rotarod performance and CD31 or endomucin mean intensity measurements. One-way ANOVA with Dunnett’s multiple comparison test; *n* = 4 (3 images per sample). *P < 0.05, ***P < 0.001, ****P < 0.0001.

Expression of markers relating to cartilage tissue integrity and inflammation were also analyzed. Noggin expression was increased in Capsaicin (95% CI of difference [31.85, 67.16], *P* < 0.0001), TMC hydrogel (95% CI of difference [24.68, 59.99], *P* < 0.0001), and TMC hydrogel + Capsaicin (95% CI of difference [31.67, 66.98], *P* < 0.0001) treatment groups compared to the MIA group, suggesting a protective effect on cartilage integrity (Fig. 7B). IL-6 expression, which is indicative of a heightened inflammatory response, was elevated in the MIA group relative to healthy control (95% CI of difference [−42.82, −11.48], *P* = 0.0007). Among the treatments, only the TMC hydrogel combined with capsaicin (95% CI of difference [−33.13, −1.799], *P* = 0.0261) effectively reduced IL-6 levels, highlighting its anti-inflammatory effects. These findings indicate that the combination therapy not only controls inflammation but also may promote structural remodeling by preserving chondrogenic signaling. However, correlation analyses revealed only a very weak positive association between Noggin expression and functional performance, whereas IL-6 showed a negative correlation (Fig. 7C).

**Fig. 7.**
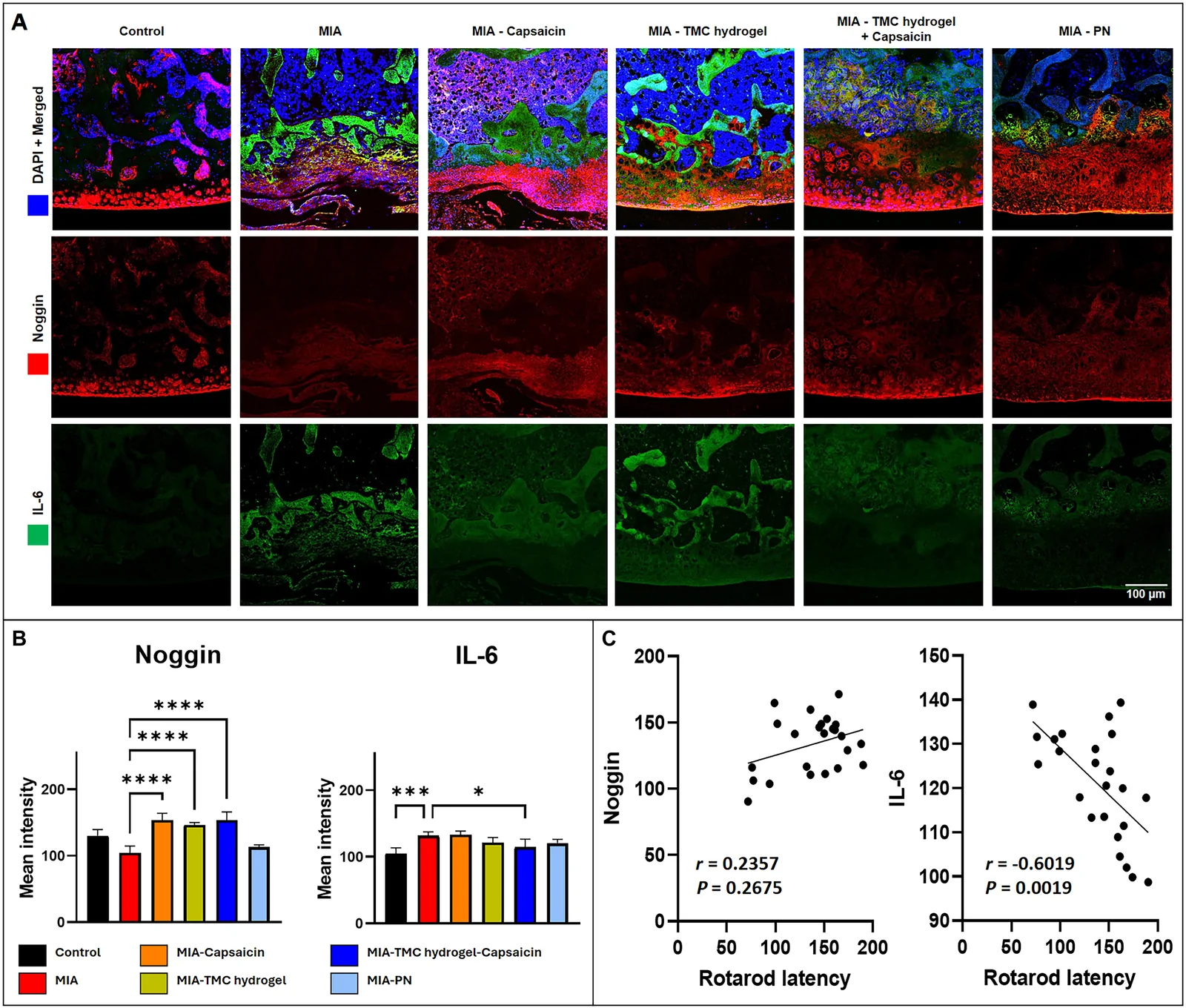
(A) Micrographs of tissue sections stained for Noggin and IL-6 to observe the effect of the different treatments on inflammatory condition and cartilage integrity. (B) Mean intensity measurements of Noggin and IL-6 from confocal images. (C) Pearson’s correlation analyses between the rotarod performance and Noggin or IL-6 mean intensity measurements. One-way ANOVA with Dunnett’s multiple comparison test; *n* = 4 (3 images per sample). *P < 0.05, ***P < 0.001, ****P < 0.0001.

## Discussion

Previously, OA was considered as a consequence of cartilage deterioration. However, as our understanding of its underlying mechanisms has evolved, it has become clear that OA is not merely a disease caused by cartilage deterioration, but rather a complex structural degradation of cartilage and other periarticular tissues. Accordingly, recent therapeutic approaches have focused on targeting the intra-articular environment [22,23].

Hydrogels have emerged as promising biomaterials for OA therapy due to their ability to provide localized, sustained drug release while maintaining biocompatibility and mechanical stability. In this study, we utilized a TMC-based thermosetting hydrogel, which we have previously utilized as a carrier for stem cells [17], and combined it for localized intra-articular delivery of capsaicin. As established and thoroughly characterized in our previous publication [17], this specific TMC hydrogel formulation is designed to degrade within approximately 1 week. Rather than acting as a permanent mechanical implant, the hydrogel was strategically engineered as a transient, biocompatible delivery vehicle. Its primary purpose is to provide a controlled, localized release of capsaicin and utilize the initial anti-inflammatory properties of the matrix without causing chronic material accumulation or foreign-body responses in the joint space. During the formulation optimization, the concentration of the organic solvent was highly considered not only for cytocompatibility but also for its structural impact on the hydrogel matrix. Thus, we opted to select the lowest possible concentration of DMSO (0.5%). This trace amount structurally benefits the hydrogel by subtly altering the water-binding network around the TMC’s quaternary ammonium groups and reducing inter-chain repulsion [10,24]. These effects were demonstrated by the absence of any observable difference in viscosity behavior between the bare and capsaicin-loaded TMC hydrogels. The intra-articular therapeutic efficacy of capsaicin is heavily dependent on its release profile, as rapid clearance or burst release can increase acute tissue irritation and limit long-term joint protection. The results of the cumulative capsaicin release revealed distinct concentration-dependent kinetics. The earlier peak observed in the higher concentration group (500 μM) can be attributed to the strong concentration gradient that rapidly pushed out surface-bound capsaicin [25,26]. In contrast, the 50 μM formulation reached the highest fractional release by the end of the observation period, likely because the lower concentration allowed capsaicin to disperse more readily as single molecules as the TMC hydrogel eroded [27].

Results of the cell viability for macrophage and rat articular cartilage cells showed that the formulation incorporating capsaicin with the TMC hydrogel did not compromise localized solvent or drug toxicity. The optimized capsaicin loading within the TMC hydrogel effectively reduced toxicity, enabling chondrocyte and macrophage tolerance up to 250 μM. This threshold is substantially higher compared to previous studies tested with different cell types [28,29]. Furthermore, IF staining and subsequent positive cell count for CD86 (M1 marker) and CD206 (M2 marker) indicated that the combination of TMC and capsaicin was accompanied by a greater reduction of proinflammatory marker expression in LPS-activated macrophage cells. Capsaicin, through TRPV1 receptor activation, is known to suppress proinflammatory M1 macrophages while promoting anti-inflammatory M2 phenotypes [30,31]. Although this is consistent with the proposed TRPV1-mediated mechanisms reported in other studies [30,31], the current study did not include TRPV1 antagonists or receptor blocking experiments, hence limiting the confirmation of TRPV1-dependent macrophage polarization. Nevertheless, when combined with the TMC hydrogel, the suppressive effect on M1 marker expression appeared to be amplified, dampening LPS-induced expression of proinflammatory markers. This may be attributed to the innate anti-inflammatory property of TMC [9,32,33]. Thus, the hydrogel not only functions as a carrier for the capsaicin but also is associated with an anti-inflammatory microenvironment conducive to cartilage tissue preservation [33–35]. This level of de-escalation toward M1 activation is a crucial factor when considering the inflammatory environment of the OA joint in which prevalent catabolic signaling drives cartilage tissue erosion and pathological joint remodeling [31].

Functional assessment using the rotarod test identified the appropriate intervention point in the OA animal model. Rotarod test for motor coordination and weight-bearing capability was used to track the dynamic physiological changes and pain relief across the experimental timeline. While automated gait analysis is a common preclinical tool to assess static parameters, it often falls short of capturing the complex deficits induced by OA pain; thus, a forced ambulatory test would provide a more rigorous challenge to the afflicted joint. Previous studies have already noted the utility of the rotarod test as a functional test for OA [36–38]. Rotarod tests motor coordination, balance, and endurance, which can be directly impacted if there is presence of articular cartilage degeneration and pain. Consequently, it provides a functional measure that closely reflects the movement-induced pain experienced in daily activities. Additionally, most diagnoses of OA are confirmed through presentation of symptomatic pain by patients [39–41], and rotarod performance provides a clinically relevant measure for evaluating injectable treatments.

A particular notable finding in our study was the divergent outcome observed in the free capsaicin treatment group, which demonstrated considerable functional improvements on the rotarod test but failed to protect the underlying cartilage tissue matrix. Results indicate that intra-articular injection of capsaicin led to mitigation of pain in short term but did not contribute to overall maintenance of the cartilage tissue integrity. These phenomena can be explained by the contrasting kinetics and localized action of unencapsulated capsaicin. When injected freely into the joint cavity, capsaicin acts as a rapid and potent agonist for TRPV1 pain receptors. This immediately triggers overstimulation followed by prolonged desensitization, which leads to effective hindrance of transmission of pain signals along the central nervous system [42]. When administered without a stabilizing vehicle such as TMC hydrogel, capsaicin undergoes rapid clearance, resulting in short-lived retention within the joint space [43]. While the transient desensitization provides pain relief, it does not reside long enough to counteract the catabolic cascade that leads to tissue degeneration. Rapid pain relief can lead the animal to place destructive mechanical stress on the compromised joint, accelerating wear and tear [44].

This critical limitation of free drug delivery highlights the potential advantage of incorporating the thermoresponsive TMC hydrogel. The TMC hydrogel appears to function as an anti-inflammatory carrier capable of sustained localized release of capsaicin in the joint cavity at a stable nontoxic concentration, which may help link immediate pain relief with longer-term tissue preservation. This interpretation is supported by the histopathological findings in which there was pronounced reduction in the averaged cartilage degradation grade for the treatments containing the TMC hydrogel, consistent with reduced severity of classic OA hallmarks such as proteoglycan depletion and aberrant chondrocyte clustering. Although all treatments resulted in improved rotarod performance, the TMC hydrogel with capsaicin treatment performed relatively more consistently compared to all other groups. These findings emphasize the importance of matrix-assisted application of capsaicin in which the hydrogel potentially influenced the bioactivity of capsaicin, contributing to the desired tissue response. Conjuran (PN) was included in this study as a clinically available intra-articular comparator commonly used for knee OA management, acting as a real-world benchmark for the functional and inflammatory outcomes. Its PN–hyaluronic acid primarily functions through scaffolding and viscosupplementation, a mechanism distinct from the pain receptor-targeted approach of the TMC hydrogel loaded with capsaicin. Hence, the comparison between these 2 treatments should be interpreted as a preclinical performance benchmark rather than as evidence of mechanistic equivalence. Any variation or similarities in the outcomes cannot be directly attributed to a shared biological phenomenon.

Previous studies have linked vascularization and bone formation as key points not only for bone regeneration but also in degenerative disease such as OA [45–47]. Aberrant angiogenesis along articular cartilage contributes to OA progression by actively supporting tissue calcification, leading to the formation of bone spurs in damaged joint tissues [48,49]. Thus, observing expression of angiogenic and osteogenic factors can further denote the level of OA progression within a joint tissue [45,50]. Treatment with the TMC hydrogel loaded with capsaicin was correlated with changes in the expression of vascular and osteogenic markers, suggesting a plausible influence on bone–cartilage crosstalk relevant to OA pathology. The observed reduction of localized CD31 and endomucin expression, alongside the increased Noggin expression along the cartilage region, suggests possible disruption of angiogenic–osteogenic coupling typically present in OA progression.

This study suggests that the application of the TMC hydrogel loaded with capsaicin is associated with improved joint function and preservation of articular cartilage in an MIA-induced OA animal model. Nevertheless, the current study is limited by the length of observation; thus, further experimentation is required to determine the effects of higher doses in later stages of OA, repeated dosing, and long-term application for single and multiple doses. Given that capsaicin is a known TRPV1 agonist, the current study does not include blockers for downstream signaling; hence, TRPV1-independent pathways or crosstalk with cationic TMC matrix cannot be entirely ruled out and will require future studies. This therapeutic strategy demonstrated promising results in this preclinical model and may warrant further investigation as a potential alternative to current intra-articular treatments for symptomatic OA, pending additional mechanistic validation and long-term safety studies.

## Data Availability

Data will be made available on request.
