# Supplementary material for: Injectable Trimethyl Chitosan Hydrogel with Capsaicin Mitigates Functional Pain and Angio-Osteogenic Coupling in an Animal Model of Osteoarthritis
Source: Biomater Res. 2026 Oct 29;30:0424. doi: 10.34133/bmr.0424 (PMC13716988; doi:10.34133/bmr.0424)
Supplement: Supplementary file 1 [file bmr.0424.f1.docx]

Supplementary 1. Weight monitoring of SD rats throughout the experiment duration.

Supplementary 2. Timed screen captures of representative rats from MIA, CAPSAICIN, TMC HYDROGEL, and TMC HYDROGEL + CAPSAICIN groups during a rotarod trial 4 weeks after treatment.
